# Modeling and Analysis of Mixed Traffic Flow Considering Driver Stochasticity and CAV Connectivity Uncertainty

**DOI:** 10.3390/s25092806

**Published:** 2025-04-29

**Authors:** Qi Zeng, Siyuan Hao, Nale Zhao, Ruiche Liu

**Affiliations:** 1 College of Mechanical and Vehicle Engineering, Hunan University, Changsha 410082, China; 15102019975@163.com; 2 Research Institute of Highway, Key Laboratory of Road Safety Technologies, Ministry of Transport, Beijing 100088, China; nl.zhao@rioh.cn (N.Z.); ruicheliu@126.com (R.L.)

**Keywords:** mixed traffic flow, driver stochasticity, connectivity uncertainties, car-following model

## Abstract

As connected and autonomous vehicle (CAV) technologies are rapidly integrated into modern transportation systems, understanding the dynamics of mixed traffic flow involving both human-driven vehicles (HVs) and CAVs is becoming increasingly important, particularly under uncertain conditions. In this paper, we propose a car-following model framework to investigate the combined effects of driver stochasticity and connectivity uncertainties of CAVs on mixed traffic flow. The proposed framework can capture the inherent stochastic variations in human driving behavior by extending the classic intelligent driver model (IDM) with a Langevin-type stochastic differential equation. A car-following model with multi-anticipation control is developed for CAVs, explicitly incorporating sensor noise, communication delays, and dynamic connectivity. Extensive numerical simulations demonstrate that higher CAV penetration leads to more stable traffic flows. Even with certain levels of connectivity uncertainty, CAVs can still effectively stabilize the traffic. However, driver stochasticity has a pronounced impact on traffic stability—greater variability in driver behavior tends to reduce overall stability. Furthermore, sensitivity analyses reveal that in pure CAV environments, sensor noise, communication delays and communication ranges can affect traffic stability and energy consumption. In contrast, in mixed traffic conditions, the inherent instability of HV behavior tends to dominate and diminish the relative influence of CAV connectivity-related uncertainties. These findings underscore the necessity of robust sensor fusion and error compensation strategies to fully realize the potential of CAV technology. In mixed traffic environments, measures should be taken to minimize the adverse effects of HVs on CAV performance.

## 1. Introduction

The rapid rise of connected and autonomous vehicle (CAV) technology is reshaping the modern transportation system. Unlike traditional human-driven vehicles (HVs) based on the driver’s subjective perception and manual control, CAVs operate through on-board sensors and communication modules, enabling more precise real-time decision-making. In the transition to fully autonomous transportation, road networks will inevitably consist of mixed traffic flows, including HVs and CAVs [1]. Understanding and accurately simulating mixed traffic behavior is critical to improving traffic management, increasing safety, and optimizing overall system performance [2,3].

Car-following behavior, which reflects the interdependence between adjacent vehicles within the same lane, is a typical microscopic driving behavior in traffic flow [4]. Despite considerable progress in car-following models, ranging from classical frameworks such as the Intelligent Driver Model (IDM) [5], the Full Velocity Difference (FVD) model [6] and their extensions [7,8,9,10] to more recent data-driven or machine learning approaches [11,12,13,14], most studies overlook two critical realities of mixed traffic. First, human driver behavior exhibits inherent stochasticity, due to interdriver heterogeneity in reaction times, risk preferences, and attention levels [15,16,17]. This randomness can amplify traffic oscillations or trigger stop-and-go waves under congested conditions. Second, although CAVs have the potential to stabilize traffic, their performance is highly dependent on connectivity reliability, which can be compromised by sensor noise, communication latency, and dynamic communication topology. Adverse weather or partial occlusion may degrade local sensor accuracy; variable wireless coverage, signal interference, or line-of-sight constraints can induce packet loss or delays [18,19]. As a result, the anticipated benefits of precise autonomous control may be diminished when these factors arise in tandem with HV-induced disturbances.

In mixed traffic environments, the interaction between the stochasticity of drivers and the uncertainty of CAV connectivity becomes particularly important. Even if CAVs use sophisticated control algorithms, the unpredictable behavior of HVs can cause disruptions or trigger safety-critical events [20]. At the same time, CAVs can use vehicle-to-vehicle (V2V) and vehicle-to-infrastructure (V2I) communications to obtain information from multiple vehicles ahead, thereby improving traffic stability and reducing collisions—provided that connectivity remains reliable [21]. This duality highlights the need for an integrated modeling approach that simultaneously handles the stochasticity of HVs and the connectivity uncertainty of CAVs to gain a deep understanding of how each aspect affects the overall traffic dynamics.

To address these challenges, this paper proposes a car-following modeling framework that combines the stochastic driver behavior of HVs and the connectivity uncertainty of CAVs. We adopt a stochastic car-following model of HVs to mathematically represent the stochastic perception mechanism of drivers by synergistically integrating IDM-based Langevin-type stochastic differential equations with an extended Cox–Ingersoll–Ross (CIR) stochastic process. In the CAV sub-model, on-board sensing and communication reliability are treated as different factors to capture the real-time changes in measurement fidelity and network connectivity. By combining these sub-models into a hybrid traffic framework, we can analyze how different CAV penetrations and different sensing and communication reliability levels affect traffic stability, throughput, and energy consumption (EC).

The main contributions of this study are threefold: First, we incorporate stochastic driver behavior into the classic car-following model to better capture the uncertainty induced by HVs. Second, we propose a car-following model for CAVs with multi-anticipation control, which incorporates sensor noise, communication delays, and dynamic connectivity. Third, through numerical simulations and sensitivity analysis, we quantify the individual and combined impacts of driver stochasticity and connection reliability on key traffic performance metrics at varying traffic densities and CAV penetrations.

The remainder of this paper is organized as follows: In Section 2, we briefly review the relevant literature and point out the existing research gaps. Section 3 introduces the mixed traffic car-following model as well as the methods for evaluating traffic stability and EC. Section 4 explains the simulation design and presents numerical results. Finally, Section 5 summarizes the main insights of the paper and potential avenues for further research.

## 2. Literature Review

### 2.1. Mixed Traffic Flow Car-Following Models

In recent years, research on mixed traffic flows consisting of HVs and CAVs has increased significantly due to the practical need to understand and optimize the coexistence of both types of vehicles. Earlier studies often explored how increasing the level of connectivity and automation in the vehicle platoon would affect overall traffic characteristics. For example, Wang et al. [22] studied a scenario where the performance of connected adaptive cruise control (CACC) was partially degraded by HV interaction and found that such mixed conditions changed the efficiency of the traffic flow. Similarly, the study by Yao et al. [23] showed how the combination of CACC vehicles with HVs affects a wider range of safety factors.

In addition to these initial explorations, many studies have improved car-following models to improve traffic stability and optimize HV-CAV interactions under different operating conditions. For example, Yao et al. [24] used the FVD model to represent HV behavior while using the CACC model to represent CAVs behavior, reflecting the different control characteristics of each vehicle type. In contrast, Xie et al. [25] developed a generalized car-following model for HVs and connected vehicles (CVs), emphasizing that the scope of information accessible to each vehicle is different. Jin et al. [26] also considered the ability of CAVs to utilize multi-vehicle data and incorporate human reaction delays, thereby providing a more unified approach to mixed traffic modeling. Other studies, such as Zhu and Zhang [27], focus on alleviating traffic congestion through novel CAV control strategies, showing that the effective implementation of such strategies can have a positive impact on traffic flow.

Some researchers have also studied operational bottlenecks such as ramp merging or lane changing. Rios-Torres and Malikopoulos [28] developed a micro-simulation framework to evaluate the interaction between HVs and CAVs in merging scenarios, thereby gaining insights into traffic performance at different levels of connectivity. Markov chain-based methods capture more random or capacity-oriented aspects of mixed traffic. For example, Ghiasi et al. [29] studied how random platooning of CAVs affects spacing distribution and road utilization. An et al. [30] further studied how real-time changes in CAV speed affect overall traffic, revealing that the dynamic actions of CAVs change key traffic parameters such as throughput or density.

Meanwhile, some studies emphasize cooperative control strategies aimed at maximizing the benefits of connectivity and mitigating the adverse effects of HV-induced disturbances. For example, Xie et al. [31] proposed a cooperative driving strategy for CVs, showing that active cooperation between CAVs can mitigate the adverse effects of unpredictable HV driving behaviors. Li et al. [32] extended a generalized model with multiple acceleration feedback loops for CAVs and conventional HVs, finding that well-chosen feedback gains can improve traffic smoothness.

### 2.2. Driver Stochasticity in Human-Driven Vehicles

It is widely believed that driver stochasticity is the main cause of traffic fluctuations. In a series of large-scale field experiments, Jiang et al. [33,34,35] demonstrated that human drivers’ choice of headways can produce significant fluctuations even when vehicles are traveling at the same speed. Their results challenge the traditional car-following assumption that acceleration and headway distance are uniquely correlated, suggesting instead that drivers’ expected headway distances may be random. Furthermore, they observed that the standard deviation of speed grows concavely along the platoon, a phenomenon that traditional car-following models cannot reproduce. These insights have motivated efforts to design random car-following models that better capture the effects of driver stochasticity on traffic flow.

To relax the one-to-one relationship between speed and spacing, Jiang et al. [35] further modeled the perceived spacing as a random variable and proposed a two-dimensional car-following model. Their framework successfully reproduced the concave growth of speed variance in empirical data. Laval et al. [36] took a more concise approach by introducing white noise into the expected acceleration term under free-flow conditions; their study showed that such random perturbations can produce traffic oscillations consistent with actual measurements. Yuan et al. [37] established a geometric Brownian motion car-following model that captured the capacity decay pattern consistent with empirical observations.

In addition, Tian et al. [38] found that speed differences usually follow a mean reversion process and proposed a random car following model with mode switching behavior. In this regard, they established a simpler random model based on Newell’s formula [39], focusing on the propagation of traffic flow waves from a longitudinal perspective. Treiber et al. [40] took a broader perspective and proposed a so-called “minimal” model that takes into account the driver’s perception threshold and randomness, thereby studying the combined impact of these factors on traffic stability.

Meanwhile, Ngoduy et al. [41] introduced an extended CIR process to describe the driver’s acceleration and derived the stability conditions of the random car following model for the first time. Ngoduy [42] embedded driver stochasticity into a high-order continuous traffic flow model from a macroscopic perspective and analyzed how noise affects the stability of traffic flow at the overall level. Bouadi et al. [43] further extended Ngoduy’s framework and provided a more robust method for evaluating the stability of random traffic flows.

### 2.3. Sensing and Communication Reliability in CAVs

In mixed traffic scenarios, the performance of CAVs hinges on not only their autonomous control algorithms but also the reliability of real-time sensing and communication. Early research efforts in this area highlighted the potential and challenges of V2V and V2I for cooperative driving strategies. Jia and Ngoduy [44] developed an enhanced car-following model, examining how packet loss, transmission delays, and measurement errors degrade traffic efficiency in cooperative systems. Similarly, Navas et al. [45] explored how CACC performance deteriorates in the absence of reliable V2V information from the immediate leader, proposing an “Advanced CACC” (ACACC) approach that capitalizes on data from farther upstream vehicles. Wang et al. [46] further stressed the importance of communication abilities by studying controllability and control design in a ring-road setting; although their focus was on the system-level impact of CAVs, they underscored that imperfect or partial connectivity introduces obstacles to fully optimizing the flow.

Beyond connectivity itself, CAVs rely heavily on local sensing—a process vulnerable to noise, measurement inaccuracies, and occlusions, particularly under adverse environmental conditions. Li et al. [47] highlighted how deviations between measured and true velocity or headway can trigger instability, influencing not just the host vehicle’s longitudinal control but also the traffic flow at large. In an overarching review, Sarker et al. [48] detailed how sensor and communication technologies, along with human factors, coalesce in an information-aware CAV controller, emphasizing that errors in sensing or transmission can profoundly affect safety and system efficiency. Shan et al. [49] demonstrated similar concerns from a “cooperative perception” perspective, where the merging of onboard sensor data and infrastructure-based detection is essential for safe autonomous maneuvers around pedestrians. Such cooperative services, though beneficial, depend on consistent and accurate data streams.

Research has also examined how to balance data accuracy with bandwidth constraints or positioning uncertainty. For instance, Tak and Choi [50] proposed a safety monitoring system that selects the optimal sampling interval for sensor data while managing the trade-off between communication overhead and data reliability. Williams et al. [51] studied the effect of positioning inaccuracies on various CAV applications, finding that even small deviations can undermine advanced functions like high-speed warning systems. Luo et al. [52] introduced a cooperative control method for CAV platoons, specifically targeting sensor measurement errors to maintain stable and comfortable longitudinal control.

## 3. Methodology

This section introduces our modeling approach for a mixed traffic flow of HVs and CAVs. First, we describe how stochastic driver behavior is incorporated into HV dynamics. We then present a car-following model with multi-anticipation control is developed for CAVs, explicitly incorporating sensor noise, communication delays, and dynamic connectivity.

**Figure 1:**
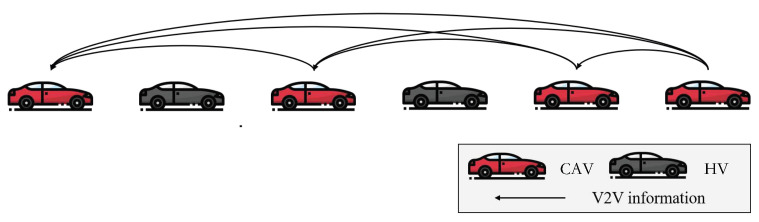
Illustration of mixed traffic flow.

As shown in Figure 1, HVs are conventional vehicles operated by human drivers without any communication capabilities, whereas CAVs can receive information from multiple preceding vehicles within their communication range.

### 3.1. Car-Following Model for HVs

In this study, we employ a stochastic car-following model for HVs by extending the IDM. Specifically, we integrate a Langevin-type stochastic differential equation to capture the variability in driver perception and response. Empirical studies, such as Jiang et al. [35], have demonstrated that human driving behavior exhibits stronger randomness at higher speed. To reflect this behavioral pattern, we adopt a velocity-dependent noise term, allowing the stochasticity to grow with speed. This approach follows the concept of an extended CIR process, which allows the random driving behavior to depend on current velocity, thus more realistically reflecting human driver heterogeneity [53]. The acceleration of vehicle *n* at time *t* is decomposed into a deterministic IDM term and a velocity-dependent noise term:

(1) $$dv_{n}\left(t\right)=a\left[1-{\left(\frac{v_{n}\left(t\right)}{v_{0}}\right)}^{\delta }-{\left(\frac{s^{*}\left(v_{n}\left(t\right),\Delta v_{n}\left(t\right)\right)}{s_{n}\left(t\right)}\right)}^{2}\right]+\sigma \sqrt{v_{n}\left(t\right)}dW_{n}\left(t\right).$$

where $v_{n}\left(t\right)$ is the velocity of the *n*-th vehicle at time *t*; *a* is the maximum acceleration; $v_{0}$ is the desired free-flow speed; δ is the acceleration exponent in IDM; $s_{n}$ denotes the pace headway between veicle *n* and its preceding vehicle; $\Delta v_{n}$ is the relative speed between vehicle *n* and its preceding vehicle; σ is the diffusion coefficient governing the strength of the velocity-dependent stochastic noise; $dW_{n}$ represents an increment of a standard Wiener process, capturing random driver fluctuations. This stochastic extension, based on an extended CIR process, captures observed variations in driver behavior, with higher speeds generally exhibiting greater acceleration randomness.

The desired spacing function $s^{*}$ within the IDM framework is given by [5]:

(2) $$s^{*}=s_{0}+Tv_{n}\left(t\right)+\frac{v_{n}\left(t\right)\Delta v_{n}\left(t\right)}{2\sqrt{ab}},$$

where $s_{0}$ is the minimum jam distance; *T* is the preferred time headway; and *b* is the comfortable deceleration rate.

Hence, the stochastic IDM in Equations (1) and (2) can be viewed as a Langevin equation where the deterministic part follows IDM logic, and the diffusion term models random driver behavior as velocity-dependent noise.

### 3.2. Car-Following Model for CAVs

In contrast to HVs, CAVs can acquire information from multiple vehicles ahead by leveraging both on-board sensors and wireless connectivity. The car-following behavior of CAVs is modeled through an extended IDM with multi-anticipation control that explicitly incorporates sensor inaccuracies, communication latency, and dynamic connectivity.

CAVs perceive their immediate environment through on-board sensors with additive Gaussian noise:

(3) $$\begin{array}{cc} \Delta {\tilde{v}}_{n}\left(t\right) & =\Delta v_{n}\left(t\right)+\epsilon_{\Delta v},\;\epsilon_{\Delta v}∼N\left(0,\sigma_{\Delta v}^{2}\right),\\ {\tilde{s}}_{n}\left(t\right) & =s_{n}\left(t\right)+\epsilon_{s},\;\epsilon_{s}∼N\left(0,\sigma_{s}^{2}\right), \end{array}$$

where ${\Delta \tilde{v}}_{n}$ and ${\tilde{s}}_{n}$ represent noisy measurements of speed difference and spacing, respectively.

Information from upstream vehicles (n−k) experiences exponentially distributed transmission delays:

(4) $$I_{n-k}^{\mathrm{recv}}\left(t\right)=I_{n-k}\left(t-\tau_{k}\right),$$

where $I_{n-k}^{\mathrm{recv}}$ represents the received information from vehicle n−k, which includes the relative speed $\Delta v_{n-k}^{\mathrm{recv}}$ and the headway $s_{n-k}^{\mathrm{recv}}$. The communication delay is denoted by $\tau_{k}∼\mathrm{Exp}\left(\lambda_{\tau }\right)$, where $\lambda_{\tau }$ is the average communication delay.

The set of connected CAVs N(t) is determined based on inter-vehicle distance:

(5) $$N\left(t\right)=\left\{n-k|d_{n,n-k}\left(t\right)\leq L_{\mathrm{range}}\right\},$$

where $d_{n,n-k}\left(t\right)$ is the absolute distance between vehicle *n* and vehicle n−k, $L_{\mathrm{range}}$ represents the effective communication range.

Following the IDM structure, the CAV car-following model integrates noisy measurements, delayed upstream data, and dynamic connectivity with a multi-anticipation control mechanism. The governing equation is given as follows:

(6) $$dv_{n}\left(t\right)=a\left[1-{\left(\frac{v_{n}\left(t\right)}{v_{0}}\right)}^{\delta }-{\left(\frac{s_{c}^{*}\left(v_{n}\left(t\right),\Delta {\tilde{v}}_{n}\left(t\right),{\left\{\Delta v_{n-k}^{\mathrm{recv}}\left(t\right)\right\}}_{n-k\in N(t)}\right)}{s_{n}^{c}\left(s_{n}\left(t\right),{\left\{s_{n-k}^{\mathrm{recv}}\left(t\right)\right\}}_{n-k\in N(t)}\right)}\right)}^{2}\right].$$

In this multi-anticipation setting, $s_{c}^{*}$ represents the desired space headway, derived from multiple preceding CAVs within the communication range, as shown in Equation (7). Meanwhile, $s_{n}^{c}$ represents the equivalent space headway of the vehicle platoon, accounting for multiple preceding CAVs, as defined in Equation (11). By leveraging upstream velocity and spacing data from V2V or V2I links, the CAV can respond more proactively than standard single-leader car-following models.

The desired space headway $s_{c}^{*}$ is formulated as follows:

(7) $$s_{c}^{*}=s_{0}+Tv_{n}\left(t\right)+\frac{v_{n}\left(t\right)\left[\left(1-\gamma \right)\Delta {\tilde{v}}_{n}\left(t\right)+\gamma \sum_{n-k\in N(t)}\eta_{n-k}\Delta v_{n-k}^{\mathrm{recv}}\left(t\right)\right]}{2\sqrt{ab}}.$$

Here, γ∈[0,1] is the multi-preceding vehicle weighting coefficient, balancing between the immediate leader’s influence and the combined impact of multiple upstream vehicles. A higher γ indicates greater reliance on upstream vehicles, while a lower γ prioritizes direct leader-following.

The weighting factor $\eta_{n-k}$ represents the relative influence strength of vehicle n−k in the upstream flow, ensuring that closer vehicles have a stronger impact than distant ones. The influence strength is governed by the following constraint:

(8) $$\sum_{n-k\in N(t)}\eta_{n-k}=1,$$

and the weight assignment follows an exponential decay function:

(9) $$\eta_{n-k}=\frac{e^{-\ell_{n,n-k}}}{\sum_{n-m\in N(t)}e^{-\ell_{n,n-m}}},$$

where $\ell_{n,n-k}$ represents the relative influence factor of vehicle n−k with respect to vehicle *n*. It is defined as follows:

(10) $$\ell_{n,n-k}=\frac{d_{n,n-k}}{v_{n}}.$$

This formulation ensures that closer preceding vehicles contribute more significantly to the control decision, while the impact of distant vehicles diminishes exponentially. The fraction $\frac{d_{n,n-k}}{v_{n}}$ intuitively captures the time required for vehicle *n* to reach vehicle n−k, making it a natural measure of influence strength.

The equivalent platoon space headway incorporating multiple upstream vehicles is defined as follows:

(11) $$s_{n}^{c}\left(t\right)=\left(1-\gamma \right){\tilde{s}}_{n}\left(t\right)+\gamma \sum_{n-k\in N(t)}\eta_{n-k}s_{n-k}^{\mathrm{recv}}\left(t\right).$$

### 3.3. Traffic Stability Evaluation

Traffic stability is assessed using the Speed Fluctuation Index SF, which quantifies the extent of velocity variations among vehicles in response to disturbances. A lower SF value indicates smoother traffic flow with reduced oscillations, whereas a higher SF suggests increased instability. The speed fluctuation for an individual vehicle *n* is defined as follows:

(12) $$SF_{n}=\frac{1}{T_{\mathrm{total}}}\sum_{t=1}^{T_{\mathrm{total}}}\left|v_{n}\left(t\right)-v_{n-1}\left(t\right)\right|,$$

where $SF_{n}$ represents the time-averaged absolute speed difference between vehicle *n* and its immediate predecessor vehicle n−1. Here, $T_{\mathrm{total}}$ denotes the total number of time steps in the simulation.

To obtain an aggregate measure of traffic stability among vehicles governed by the proposed model, the mean speed fluctuation is computed as follows:

(13) $$SF=\frac{1}{\left|S\right|}\sum_{n\in S}SF_{n},$$

where S denotes the set of vehicles whose motion is controlled by the proposed car-following model, and |S| is the cardinality of that set.

### 3.4. Energy Consumption Evaluation

As electrification represents the future direction of transportation systems, this study assumes that all vehicles in the mixed traffic flow are electric vehicles (EVs). The power-based EV EC model proposed by Fiori et al. [54] is adopted to evaluate EC in mixed traffic flow.

The EC model follows a backward structure. It first determines the instantaneous power at the wheels, $P_{W}\left(t\right)$, based on vehicle kinematics, vehicle characteristics, road grades, and rolling resistance. Subsequently, power transmission losses, including those from the engine, drivetrain, and battery, are accounted for to compute the traction power demand, $P_{T}\left(t\right)$, or regenerative power, $P_{R}\left(t\right)$. The integration of $P_{T}\left(t\right)$ and $P_{R}\left(t\right)$ over the entire driving cycle yields the total EC and recovery. Additionally, given an initial state of charge (SOC), its variation throughout the driving cycle can be tracked.

The instantaneous power at the wheels is formulated as follows [54]:

(14) $$P_{W}\left(t\right)=\left(ma\left(t\right)+mg\cos\left(\theta \right)\frac{C_{r}}{1000}\left(c_{1}v\left(t\right)+c_{2}\right)+\frac{1}{2}\rho_{\mathrm{air}}A_{f}C_{D}v^{2}\left(t\right)+mg\sin\left(\theta \right)\right)v\left(t\right),$$

where *m* is the vehicle mass; a(t) and v(t) represent acceleration and velocity at time *t*; *g* is the gravitational acceleration (9.8066 ${m/s}^{2}$); θ is the road grade; $C_{r}$, $c_{1}$, and $c_{2}$ are rolling resistance parameters; $\rho_{\mathrm{air}}$ is the air mass density, taken as 1.2256 ${\mathrm{kg}/m}^{3}$ at sea level at 15 °C; $A_{f}$ is the vehicle’s frontal area; and $C_{D}$ is the aerodynamic drag coefficient.

Given $P_{W}\left(t\right)$, the required traction power $P_{T}\left(t\right)$ or regenerative power $P_{R}\left(t\right)$ is determined by the sign of $P_{W}\left(t\right)$, distinguishing between power output and energy recovery:

(15) $$\left\{\begin{array}{cc} P_{T}\left(t\right)=P_{W}\left(t\right)/\left(\eta_{DL}\eta_{EM}\eta_{BAT}\right), & \mathrm{if}\;P_{W}\left(t\right)\geq 0,\\ P_{R}\left(t\right)=-P_{W}\left(t\right)\eta_{DL}\eta_{EM}\eta_{BAT}\eta_{RB}\left(t\right), & \mathrm{if}\;P_{W}\left(t\right)<0. \end{array}\right.$$

Here, $\eta_{DL}$, $\eta_{EM}$, and $\eta_{BAT}$ denote the efficiency of the driveline, electric motor, and battery system, respectively. The regenerative braking efficiency, $\eta_{RB}\left(t\right)$, depends on instantaneous acceleration and follows:

(16) $$\eta_{RB}\left(t\right)=\left\{\begin{array}{cc} e^{-\frac{\alpha }{\left|a(t)\right|}}, & \mathrm{if}\;a(t)<0,\\ 0, & \mathrm{if}\;a(t)\geq 0. \end{array}\right.$$

where α is a vehicle-specific parameter.

The total EC ($E_{\mathrm{Consumption}}$) and energy recovery ($E_{\mathrm{Recovery}}$) over a driving cycle are computed as follows:

(17) $$E_{\mathrm{Consumption}}=\int_{t_{0}}^{t_{f}}P_{T}\left(t\right)dt,\;E_{\mathrm{Recovery}}=\int_{t_{0}}^{t_{f}}P_{R}\left(t\right)dt,$$

where $t_{0}$ and $t_{f}$ denote the start and end times of the simulation. The net EC, denoted as $E_{NC}$, is expressed as follows:

(18) $$E_{NC}=E_{\mathrm{Consumption}}-E_{\mathrm{Recovery}}.$$

## 4. Numerical Experiments

To analyze the characteristics of mixed traffic flow comprising HVs and CAVs, numerical experiments are conducted on a 2000-m, single-lane road segment under open-boundary conditions. The open boundary setting allows vehicles to enter and leave the system freely, thereby emulating real-world traffic inflow and outflow. The simulation involves a heterogeneous platoon of 30 vehicles, where the leading vehicle follows a predefined velocity profile, while the subsequent vehicles adhere to the proposed car-following models. All simulations are implemented using MATLAB R2024a.

**Figure 2:**
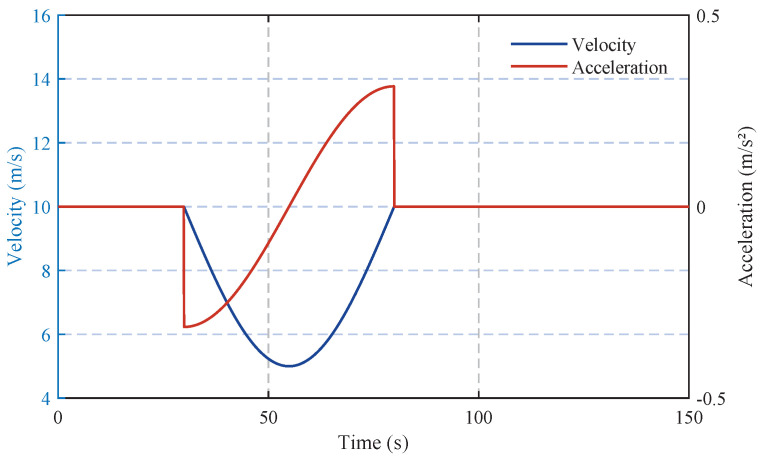
Velocity and acceleration profiles of the leading vehicle.

To examine how disturbances propagate through the traffic flow, the velocity profile of the leading vehicle incorporates a sinusoidal fluctuation, as depicted in Figure 2. This fluctuation mimics real-world driving perturbations, introducing controlled disturbances into the platoon. The key simulation parameters are set as follows: $a=2\;{m/s}^{2}$, $b=2\;{m/s}^{2}$, δ=4, $v_{0}=20\;m/s$, $s_{0}=4\;m$, and T=1.5s [53].

### 4.1. Impact of CAV Penetration Rate

**Figure 3:**
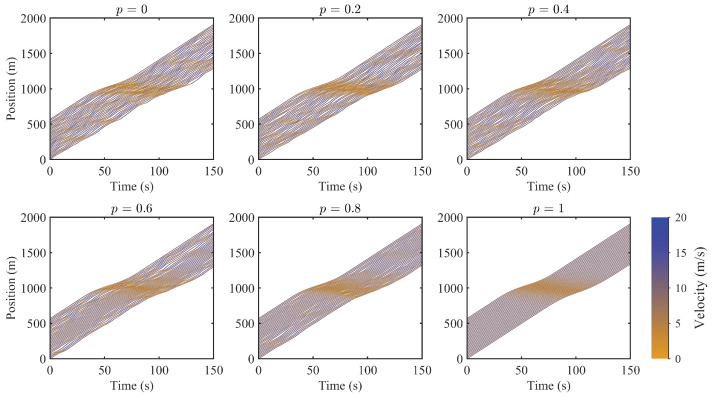
Time-space diagram of traffic flow under different CAV penetration rates.

To investigate the effect of CAVs on mixed traffic flow, simulations are performed for different CAV penetration rates. Figure 3 illustrates the spatiotemporal evolution of traffic flow as the penetration rate increases (p=0.0,0.2,0.4,0.6,0.8,1.0). The simulations are conducted with σ=0.5, $\epsilon_{\Delta v}=0.1$, $\epsilon_{s}=0.2$, $\lambda_{\tau }$ = 1.5 s, $L_{\mathrm{range}}=100$ m, and γ = 0.2. The positions of CAVs within the platoon are randomly assigned.

The results show that with increasing penetration, speed fluctuations decrease and traffic flow runs more smoothly. The higher the randomness of the drivers, the more likely stop-and-go behavior occurs, indicating unstable traffic conditions. Despite sensor errors, communication delays, and connectivity limitations, CAVs can effectively mitigate the instability caused by the randomness of human driving. Even under a given uncertainty setting, the proposed control strategy enables CAVs to suppress traffic fluctuations and significantly improve traffic flow stability.

### 4.2. Impact of Driver Stochasticity

To evaluate the impact of driver stochasticity on traffic stability and analyze how the penetration of CAVs can mitigate its effects, we conduct numerical experiments under different values of the stochastic parameter σ and consider two CAV penetration levels: p=0.2 and p=0.8.

**Figure 4:**
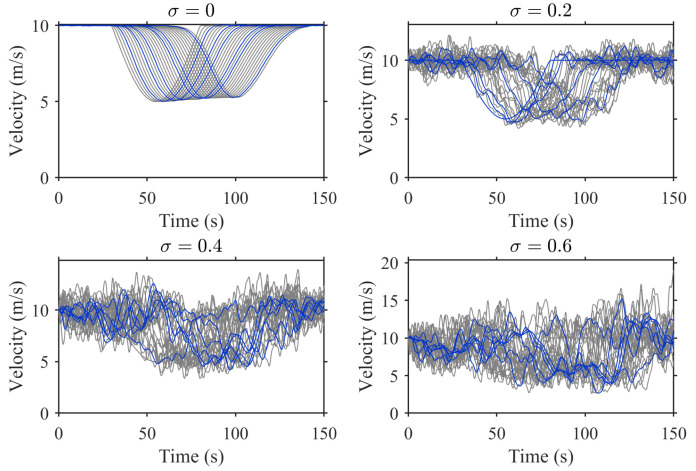
Temporal evolution of vehicle velocities under different σ values (p=0.2).

Figure 4 presents the temporal evolution of vehicle velocities under the condition of p=0.2 under different values of σ. The trajectories of HVs and CAVs are distinguished by line colors: gray for HVs and blue for CAVs. With the increase of σ, the velocity fluctuations of HVs become more obvious, resulting in oscillation amplification and frequent acceleration-deceleration cycles. These fluctuations propagate in the traffic flow, eventually forming a stop-and-go traffic pattern.

When σ=0, HVs maintain relatively stable velocity profiles. However, as σ increases, their velocity trajectories exhibit greater oscillations, indicating a significant decrease in traffic stability. In contrast, CAVs have smoother velocity profiles compared to HVs due to the adoption of cooperative driving strategies. However, their ability to suppress overall traffic fluctuations is still limited due to their relatively low penetration rate (p=0.2). Even in the case of high driver stochasticity (σ=0.6), while CAVs can alleviate some of the disturbances brought by HVs, their stabilizing effect is not enough to completely eliminate traffic fluctuations. This confirms that higher driver stochasticity reduces traffic stability, and while CAVs help offset these fluctuations, their effect is limited when penetration is low.

To quantitatively evaluate the impact of driver stochasticity and CAV penetration on traffic stability, Table 1 lists the SF calculated using Equation (13) for different σ values under p=0.2 and p=0.8. The results show a clear positive correlation between σ and SF, indicating that the higher the driver stochasticity, the greater the traffic instability.

At a low CAV penetration rate (p=0.2), the SF value rises sharply from 0.07 to 1.34 as σ increases, reflecting the significant speed fluctuations and intensified stop-and-go waves caused by human driving randomness. However, when the CAV penetration rate increases to p=0.8, the SF value remains continuously low at all σ levels. For instance, when σ=0.4, the SF is reduced by 62.8%. As σ increases, the stabilizing effect of CAVs becomes more pronounced. In the given simulation settings, increasing the CAV penetration from 0.2 to 0.8 leads to an average improvement of 46.5% in traffic stability. These results confirm that CAVs effectively mitigate the instability caused by human drivers, and their benefit grows with higher penetration. Notably, even under high driver stochasticity (σ=0.6), the presence of more CAVs can significantly reduce speed fluctuations, highlighting the potential of cooperative driving strategies in stabilizing mixed traffic flows.

### 4.3. Impact of Connectivity Uncertainty

The impact of connection uncertainty on flow stability and EC is investigated through numerical experiments. The following key factors are studied: sensor noise, communication delay and communication range. The traffic stability is quantified using SF, while EC is evaluated based on the electric vehicle powertrain model (Equations (14)–(18)), whose parameters are listed in Table 2. To ensure consistency with previous research, we adopt the Nissan Leaf as the representative EV model and use the same parameter settings as in [54,55].

#### 4.3.1. Effect of Sensor Noise

CAVs rely on sensors to measure relative speed $\Delta {\tilde{v}}_{n}$ and vehicle spacing ${\tilde{s}}_{n}$. The presence of sensor noise can lead to errors in traffic state perception and affect the accuracy of vehicle response. We analyze this effect by introducing Gaussian noise into speed and spacing measurements.

Sensor noise levels are divided into four levels, as shown in Table 3. The higher the noise level, the greater the measurement uncertainty, which leads to a decrease in CAV control performance and increased traffic instability.

**Figure 5:**
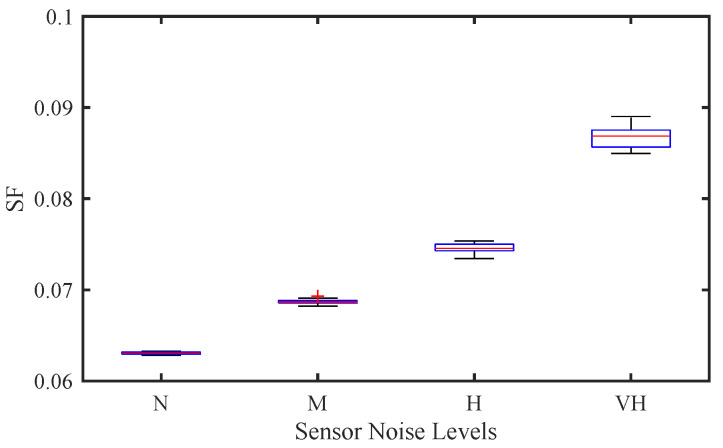
Effect of sensor noise on traffic stability of CAV traffic flow.

Figure 5 shows the effect of sensor noise level on traffic stability in a pure CAV traffic flow using SF. As sensor noise increases from N to VH, SF shows a clear upward trend, indicating a decrease in traffic stability. At the lowest noise level (N), SF remains minimal, indicating that CAVs can accurately perceive relative speed and spacing information, resulting in smooth vehicle interaction and stable traffic flow. When the noise increases to M and H, SF shows a moderate increase. This indicates that the control performance of CAVs gradually deteriorates due to the increase in measurement errors of speed and spacing, resulting in more obvious vehicle speed fluctuations. At the highest noise level (VH), SF reaches a peak, indicating severe traffic instability. The amplified fluctuations indicate that excessive sensor noise prevents CAVs from accurately responding to real-time traffic conditions, resulting in amplified stop-and-go waveforms and loss of vehicle string stability. These findings confirm that sensor noise has a negative impact on traffic stability, and higher noise levels lead to greater speed fluctuations.

**Figure 6:**
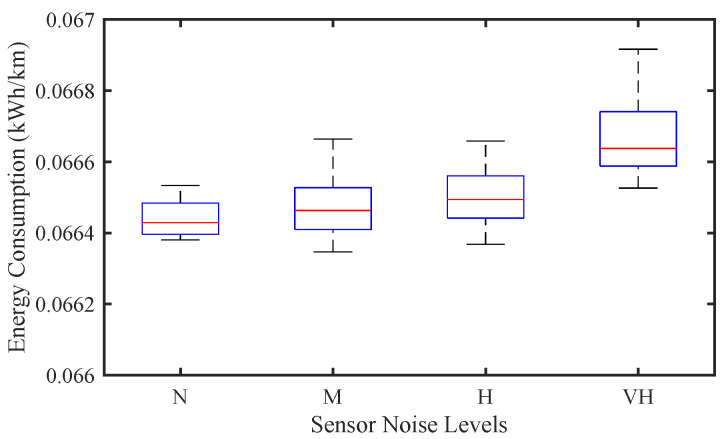
Effect of sensor noise on energy consumption of CAV traffic flow.

Figure 6 illustrates the effect of sensor noise on EC. As the noise level increases from N to VH, a gradual increase in EC can be observed. At the lowest noise level (N), EC remains relatively low and stable, indicating that the CAV can maintain a smooth acceleration and deceleration pattern, thereby reducing unnecessary EC. This suggests that the reduction in the accuracy of speed and headway measurement leads to increased control errors, resulting in more frequent speed adjustments, which in turn increase EC. At the highest noise level (VH), EC reaches a peak with a larger interquartile range, indicating a larger difference in EC between different vehicles. This increase can be attributed to the increase in speed fluctuations and more aggressive braking and acceleration events, as the CAV has difficulty responding to the distorted sensor information. These results confirm that higher sensor noise not only affects traffic stability but also leads to increased EC.

**Figure 7:**
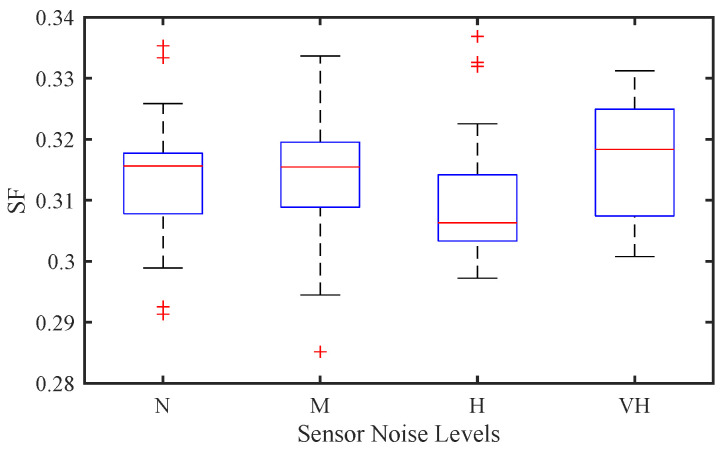
Effect of sensor noise on traffic stability of mixed traffic flow.

**Figure 8:**
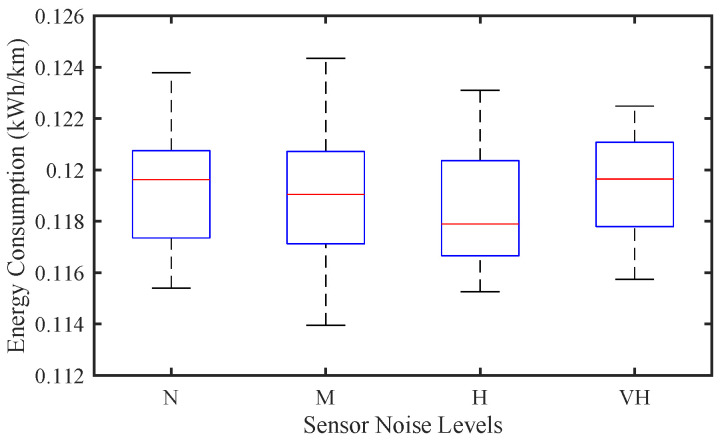
Effect of sensor noise on energy consumption of mixed traffic flow.

Figure 7 and Figure 8 illustrate the impact of sensor noise on traffic stability and EC in a mixed traffic environment, where the driver stochasticity parameter is fixed at σ=0.2 and the CAV penetration rate is set to a high level of p=0.8. Unlike in purely CAV-dominated traffic, where sensor noise tends to have a more pronounced influence, the figures reveal that the effect of sensor noise in mixed traffic is relatively minor.

From Figure 7, it can be observed that the SF exhibits only slight variations across different noise levels. The stability degradation caused by increasing sensor noise is not as significant as expected, suggesting that in mixed traffic conditions, the influence of human-driven vehicle randomness dominates over the sensor noise from CAVs. The presence of HVs introduces greater unpredictability, overshadowing the effect of sensor errors in CAVs.

Similarly, Figure 8 shows that EC remains relatively stable despite the variation in sensor noise levels. Unlike in purely CAV environments, where precise perception and control are crucial for energy efficiency, the EC in mixed traffic is predominantly dictated by the driving behaviors of human drivers, leading to a weaker correlation with CAV sensor noise levels.

These findings highlight a key implication: to fully exploit the benefits of CAVs, it is crucial to minimize interactions with HVs by implementing dedicated lanes or physically separating CAV and HV traffic flows. By reducing the disturbances introduced by human driving behavior, the advantages of CAVs in maintaining smooth and energy-efficient traffic flow can be better realized.

#### 4.3.2. Effect of Communication Delays

**Figure 9:**
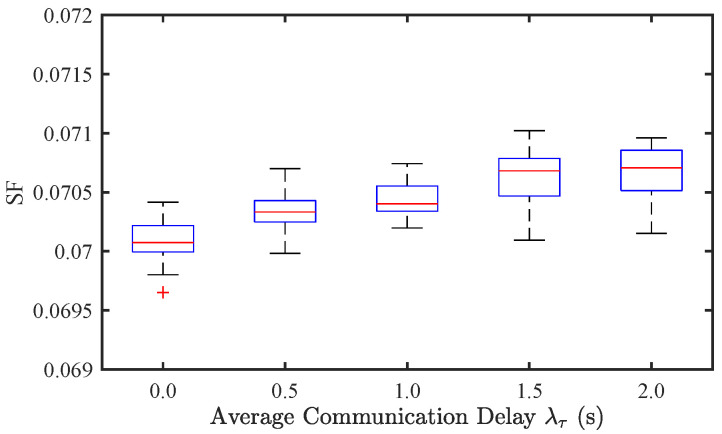
Effect of communication delay on traffic stability of CAV traffic flow.

**Figure 10:**
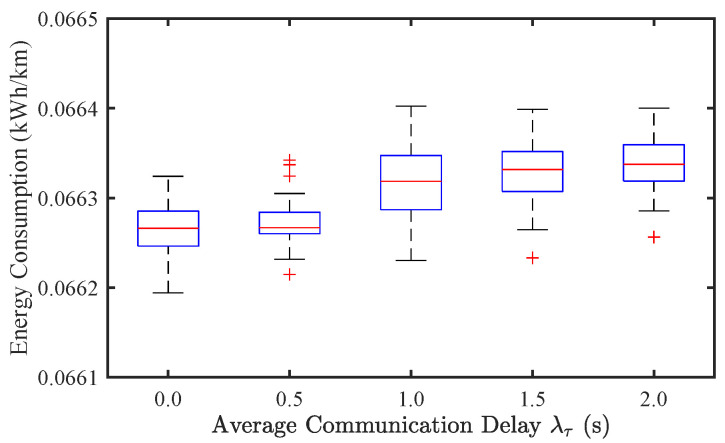
Effect of communication delay on energy consumption of CAV traffic flow.

To investigate the impact of communication delay on traffic stability and EC, we conducted simulations under different levels of average communication delay, specifically $\lambda_{\tau }=0,0.5,1,1.5,2$ s. Figure 9 and Figure 10 illustrate the results for CAV-only traffic flow. It can be observed that as $\lambda_{\tau }$ increases, the SF exhibits an increasing trend, indicating a degradation in traffic stability. Simultaneously, EC also rises due to increased velocity oscillations. However, compared to the effect of sensor noise (Figure 5 and Figure 6), the impact of communication delay appears to be less significant.

**Figure 11:**
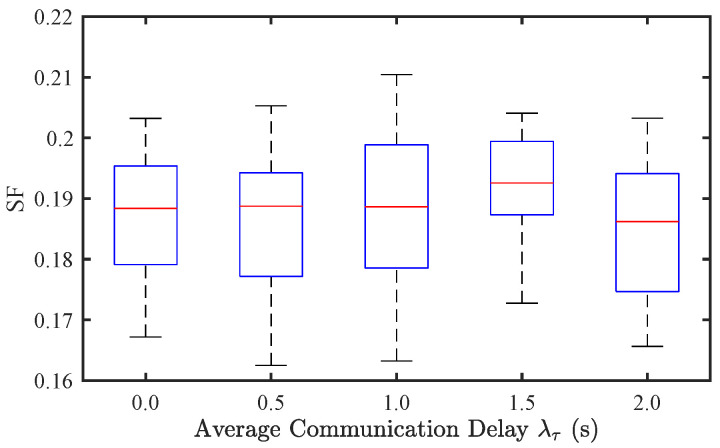
Effect of communication delay on traffic stability of mixed traffic flow.

**Figure 12:**
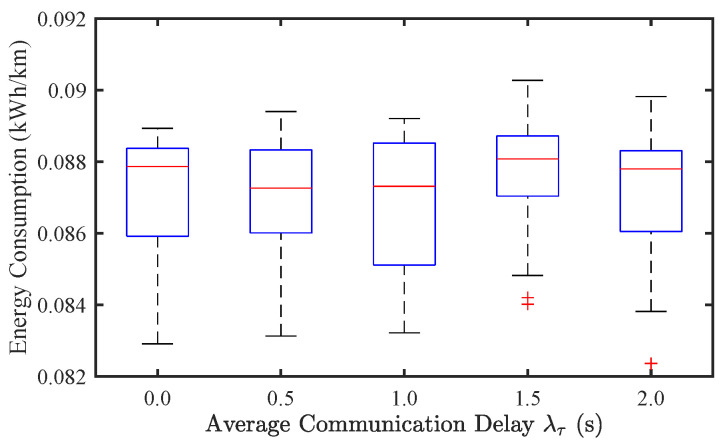
Effect of communication delay on energy consumption of mixed traffic flow.

To further explore its effect in mixed traffic conditions, we analyze communication delay in a mixed traffic environment with a CAV penetration rate of p=0.8. As shown in Figure 11 and Figure 12, the variations in SF and EC across different $\lambda_{\tau }$ values are relatively minor, aligning with the trends observed for sensor noise. This suggests that in mixed traffic flow, the impact of communication delay on stability and energy efficiency is limited. The presence of human-driven vehicles introduces greater randomness, which dominates traffic dynamics compared to communication uncertainties in CAVs.

#### 4.3.3. Effect of Communication Range

**Figure 13:**
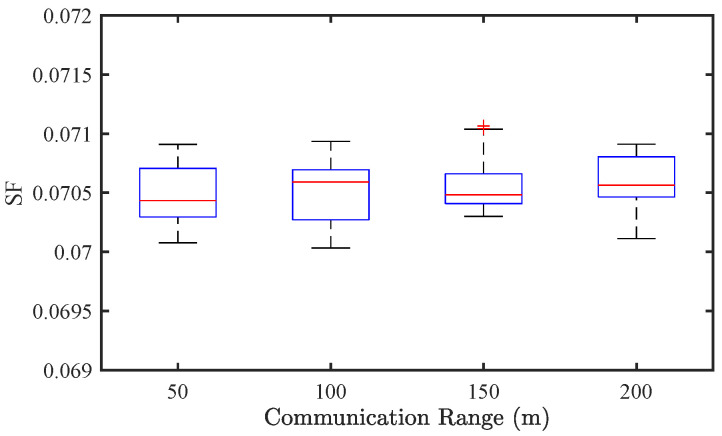
Effect of communication range on traffic stability of CAV traffic flow.

**Figure 14:**
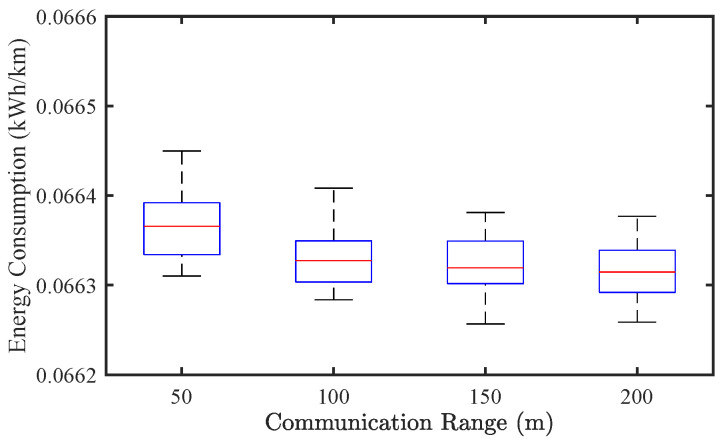
Effect of communication range on energy consumption of CAV traffic flow.

To further examine the effect of communication range on traffic performance, we conduct simulations by varying the communication range from 50 m to 200 m. Figure 13 and Figure 14 illustrate the results for CAV-only traffic flow. From the perspective of traffic stability, the SF shows minimal change (from 0.072 to 0.069) as the communication range increases, suggesting that even a relatively short communication horizon is sufficient to maintain stable flow under the given experimental setup. In terms of energy consumption, EC decreases slightly from approximately 0.0665 to 0.0663 kWh/km, indicating that extended communication range enables marginal improvements in energy efficiency.

**Figure 15:**
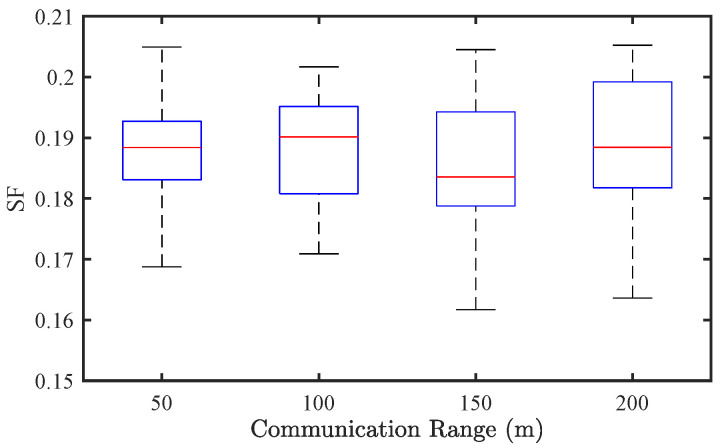
Effect of communication range on traffic stability of mixed traffic flow (p=0.8).

**Figure 16:**
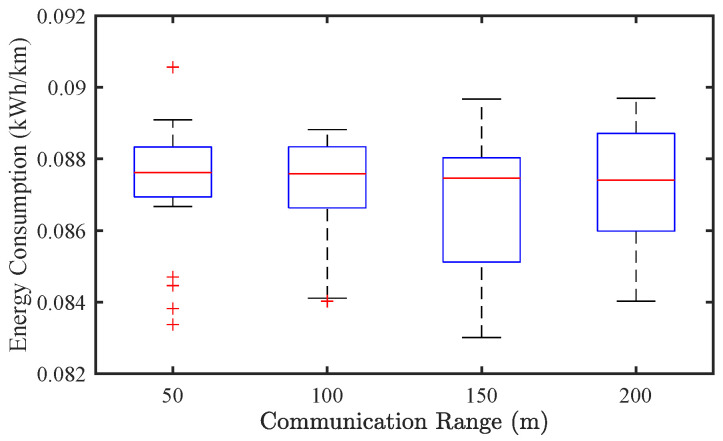
Effect of communication range on energy consumption of mixed traffic flow (p=0.8).

Figure 15 and Figure 16 present the results under a mixed traffic condition with a CAV penetration rate of p=0.8. Similar to the CAV-only case, the influence of communication range on SF remains negligible. Regarding EC, the effect of communication range is even less significant in mixed traffic, with only marginal differences observed across different settings. These findings suggest that, under the tested conditions, communication range has limited impact on system performance, especially when human driving stochasticity dominates traffic dynamics.

## 5. Conclusions

In this paper, we develop a comprehensive car-following framework that not only captures the stochastic nature of human driving behavior but also incorporates the connectivity uncertainties inherent in CAV operation. Our model is based on an extended CIR process, which extends the classical IDM with Langevin-type stochastic differential equations to effectively represent the stochastic fluctuations of driver perception and response. For CAVs, we design a multi-anticipation control strategy to account for sensor noise, communication delays, and dynamic connectivity, thereby integrating local perception and V2X communication effects into vehicle dynamics.

Extensive numerical experiments yield several important insights. The increasing penetration of CAVs generally leads to enhanced traffic stability, as evidenced by reduced speed fluctuations and smoother vehicle trajectories. In the given simulation settings, increasing the CAV penetration from 0.2 to 0.8 leads to an average improvement of 46.5% in mixed traffic stability. While high levels of driver stochasticity can lead to significant speed fluctuations and stop-and-go wave, CAVs can effectively mitigate such instabilities—even in the presence of sensor noise, communication delays, and connectivity limitations. The proposed control strategy enables CAVs to suppress traffic fluctuations and maintain flow stability under various uncertain conditions. Furthermore, sensitivity analysis shows that while sensor errors, communication delays, and communication ranges can affect traffic stability and energy consumption in fully automated driving scenarios, their impact is reduced in mixed traffic, where the stochasticity of human drivers dominates. Our results suggest that separating CAVs from HVs—for example, by using dedicated CAV lanes—can be a simple and effective way to improve traffic flow. In our simulations, CAV-only traffic is more stable and uses less energy than mixed traffic, even when there are sensor or communication issues. This is mainly because human driver randomness in mixed traffic reduces the benefits of CAV control. Some countries have already started testing dedicated CAV lanes, which shows this idea could work in practice. These results show that higher CAV use should be matched with good sensors, reliable communication, and proper road design to reap the full benefits.

Future research will focus on extending the proposed model to multi-lane and urban traffic scenarios, developing adaptive control strategies in dynamic environments, and integrating advanced filtering techniques to further improve the robustness and efficiency of automated traffic management systems. In addition, future work will focus on the empirical calibration of key parameters related to driver stochasticity, connectivity uncertainty, and especially sensor noise, to enhance the realism and practical relevance of the proposed model. 

## Figures and Tables

**Table 1:** SF under different σ values and CAV penetration rates.

σ	SF (*p* = 0.2)	SF (*p* = 0.8)
0	0.07	0.07
0.2	0.44	0.18
0.4	0.86	0.32
0.6	1.34	0.48

**Table 2:** The description and setting values of parameters for the energy consumption model.

Parameters	Descriptions	Values
m(kg)	Vehicle mass	1521
$g\;({m/s}^{2})$	Gravitational acceleration	9.8066
θ	Road grade	0
$C_{r},c_{1},c_{2}$	Rolling resistance factors	1.75, 0.0328, 4.575
$\rho_{air}\;\left({\mathrm{kg}/m}^{3}\right)$	Air mass density	1.2256
$A_{f}\;\left(m^{2}\right)$	Vehicle front area	2.3316
$C_{D}$	Vehicle’s aerodynamic drag coefficient	0.28
$\varpi_{DL}$	Driveline efficiency	0.92
$\varpi_{EM}$	Electric motor efficiency	0.91
$\varpi_{BAT}$	Battery efficiency	0.90
ϑ	A constant	0.0411

**Table 3:** Sensor noise levels for velocity and spacing measurements.

Noise Level	$\sigma_{\Delta v}$ (m/s)	$\sigma_{s}$ (m)
No noise (N)	0	0
Medium (M)	0.1	0.2
High (H)	0.2	0.4
Very High (VH)	0.4	0.8

## Data Availability

Dataset available on request from the authors.
